# Association of Vitamin K Status with Arterial Calcification and Stiffness in Chronic Kidney Disease: The Chronic Renal Insufficiency Cohort

**DOI:** 10.1016/j.cdnut.2022.100008

**Published:** 2022-12-23

**Authors:** M. Kyla Shea, Jifan Wang, Kathryn Barger, Daniel E. Weiner, Raymond R. Townsend, Harold I. Feldman, Sylvia E. Rosas, Jing Chen, Jiang He, John Flack, Bernard G. Jaar, Mayank Kansal, Sarah L. Booth, CRIC Study Investigators

**Affiliations:** 1 USDA Human Nutrition Research Center on Aging, Tufts University, Boston, MA, USA; 2 Division of Nephrology, Tufts Medical Center, Boston, MA, USA; 3 Perelman School of Medicine, University of Pennsylvania, Philadelphia, PA, USA; 4 Joslin Diabetes Center, Beth Israel Deaconess Medical Center and Harvard Medical School, Boston, MA, USA; 5 Department of Epidemiology, Tulane University School of Medicine, New Orleans, LA, USA; 6 Department of Internal Medicine, Southern Illinois University School of Medicine, Springfield, IL, USA; 7 Department of Medicine, Johns Hopkins University, Baltimore, MD, USA; 8 Department of Medicine, University of Illinois–Chicago, Chicago, IL, USA

**Keywords:** vitamin K, arterial calcification, arterial stiffness, vascular calcification, chronic kidney disease, matrix gla protein

## Abstract

**Background:**

Arterial calcification and stiffness are common in people with chronic kidney disease (CKD). Higher vitamin K status has been associated with less arterial calcification and stiffness in CKD in cross-sectional studies.

**Objectives:**

To determine the association of vitamin K status with coronary artery calcium (CAC) and arterial stiffness [pulse wave velocity (PWV)] at baseline and over 2–4 follow-up years in adults with mild-to-moderate CKD.

**Methods:**

Participants (*n* = 2722) were drawn from the well-characterized Chronic Renal Insufficiency Cohort. Two vitamin K status biomarkers, plasma phylloquinone and plasma dephospho-uncarboxylated matrix gla protein [(dp)ucMGP], were measured at baseline. CAC and PWV were measured at baseline and over 2–4 y of follow-up. Differences across vitamin K status categories in CAC prevalence, incidence, and progression (defined as ≥100 Agatston units/y increase) and PWV at baseline and over follow-up were evaluated using multivariable-adjusted generalized linear models.

**Results:**

CAC prevalence, incidence, and progression did not differ across plasma phylloquinone categories. Moreover, CAC prevalence and incidence did not differ according to plasma (dp)ucMGP concentration. Compared with participants with the highest (dp)ucMGP (≥450 pmol/L), those in the middle category (300–449 pmol/L) had a 49% lower rate of CAC progression (incidence rate ratio: 0.51; 95% CI: 0.33, 0.78). However, CAC progression did not differ between those with the lowest (<300 pmol/L) and those with the highest plasma (dp)ucMGP concentration (incidence rate ratio: 0.82; 95% CI: 0.56, 1.19). Neither vitamin K status biomarker was associated with PWV at baseline or longitudinally.

**Conclusions:**

Vitamin K status was not consistently associated with CAC or PWV in adults with mild-to-moderate CKD.

## Introduction

Chronic kidney disease (CKD) can be characterized by mineral imbalances causing calcium deposition throughout the vasculature [1, 2], which contributes to a higher risk for CVD and mortality. Vascular calcification can occur in the intimal and medial arterial layers. Intimal calcification, which is common in the coronary arteries [known as coronary artery calcification (CAC)], is a subclinical manifestation of atherosclerotic CVD. Medial calcification, which occurs more peripherally, contributes to arterial stiffening, heart failure, and atrial fibrillation [3]. CAC and arterial stiffness are common in CKD [4, 5]. Therefore, identifying therapies that reduce arterial calcification in individuals with CKD is important.

The vitamin K–dependent protein matrix gla protein (MGP) is present in arterial tissues [6]. In the presence of vitamin K, MGP becomes carboxylated. Carboxylated MGP inhibits arterial calcification, whereas uncarboxylated MGP does not [7]. A diet high in vitamin K enhanced MGP carboxylation and reduced arterial calcification and stiffness in rodents with CKD [8, 9]. Several [but not all [10]] observational studies have reported that elevated circulating uncarboxylated MGP (reflective of lower vitamin K status) was associated with more arterial calcification [11, 12] and arterial stiffening [13, 14] in CKD. However, the available studies are cross-sectional and conducted in people with end-stage kidney disease [[10], [11], [12], [13]]. Longitudinal studies of the association of vitamin K status with arterial calcification and stiffness in individuals with mild-to-moderate CKD are lacking. To address this gap, we leveraged data from the Chronic Renal Insufficiency Cohort (CRIC) and evaluated the association of plasma phylloquinone (vitamin K1) and plasma dephospho-uncarboxylated MGP [(dp)ucMGP] with CAC and aortic pulse wave velocity (PWV) cross-sectionally and over 2–4 y of follow-up. We hypothesized that lower plasma phylloquinone and higher (dp)ucMGP would be associated with more CAC and a higher PWV.

## Methods

The CRIC study is an ongoing prospective study designed to identify novel risk factors for CKD progression and CVD in patients with mild-to-moderate CKD [15, 16]. From 2003 to 2008, CRIC enrolled 3939 patients with moderate CKD from clinical centers across 7 cities in the United States. Vitamin K status biomarkers were measured from samples obtained at the 12-mo follow-up visit in 3402 CRIC participants. Of these, 2993 participants reported CAC and/or PWV measurements. We additionally excluded 166 who were taking or had missing information about the vitamin K antagonist anticoagulant warfarin and 105 with missing covariate data, leaving 2722 available for inclusion (Supplemental Figure 1). The institutional review boards of all participating centers approved the CRIC protocols, and procedures were followed according to the tenets of the Helsinki Declaration. All participants provided written informed consent, which included consent for use of data and biospecimens for future research. Additional details of the CRIC study design and methods are provided elsewhere [15].

### CAC

At the 12-mo visit, a subgroup of CRIC participants underwent a CT examination with either electron beam or multidetector CT [17]. Repeat CT scans were performed on 57% of the subgroup (*n* = 1016) at a median of 3 y later. All scans were performed by trained and certified technologists, read, and scored using the method of Agatston by a cardiologist at the central reading center, as described [17, 18]. Among those with no baseline CAC [baseline Agatston score (AS) = 0], incidence was defined as an AS >0 at the follow-up. Among those with baseline CAC (baseline AS >0), progression was defined as an annual AS increase ≥100 Agatston units (AUs) because annual increases of this magnitude are associated with an increased risk for ischemic heart disease [19, 20]. The average AS of 2 scans at each visit was used in all analyses. One participant with a −1116 AS decrease from the first to second scans was excluded from the analysis.

### PWV

PWV was measured by trained technologists using the Sphygmocor PVx System (AtCor Medical) using the right carotid and right femoral arteries, in a subgroup of CRIC participants, as described in detail [21]. Up to 4 repeat measures were obtained in 73% of the participants (*n* = 1836), over an average follow-up time of 3 y. Participants were in a supine position and rested for ≥5 min before each measurement. An adjusted PWV measure, calculated to account for the distance between the sternal notch and femoral artery and waist circumference–induced measurement [described previously [21]], was used in all analyses.

### Vitamin K status

The concentrations of (dp)ucMGP were measured using a commercially available automated sandwich ELISA, which uses 2 anti-MGP monoclonal antibodies directed against dephosphorylated uncarboxylated MGP [Immunodiagnostics Systems; interassay and intra-assay CV 4.0% and 4.5%, respectively]. The assay’s lower limit of detection (LLD) is 300 pmol/L. Higher plasma (dp)ucMGP concentration reflects lower vitamin K status. Plasma phylloquinone was measured using high-performance liquid chromatography [22]. The assay’s lower LLD is 0.1 nmol/L. The laboratory participates in the vitamin K quality assurance scheme [23], consistently generating serum/plasma phylloquinone data within the acceptable expected value range (analyses occurs every 4 mo, for >30 cycles of verification). The intra-aasay and interassay CV are 4.2% and 4.9%, respectively. All plasma samples were obtained in the fasted state and stored at −80°C until the laboratory analysis [24].

### Covariates

Sociodemographic information, including age, sex, education, race/ethnicity, and medical history information, was obtained through self-reported questionnaires. Weight and height were measured using standard protocols, and BMI was calculated as weight in kilograms divided by the square of height in meters [16]. Estimated GFR was calculated using CRIC-specific equations [25]. Diabetes mellitus was defined as a fasting plasma glucose concentration of ≥126 mg/dL, a nonfasting plasma glucose concentration of ≥200 mg/dL or self-reported use of antidiabetic medication. Blood pressure was based on 3 seated measurements that were obtained by trained staff after 5 min of rest. Hypertension was defined as mean systolic/diastolic blood pressures of ≥140/90 mm Hg or self-reported use of antihypertensive medications [26]. Triglycerides were measured using an enzymatic colorimetric assay [27]. Most of the covariate measurements were obtained at the 12-mo clinic visit, at the same visit when vitamin K status was measured. Heart rate at baseline (or the closest measure, if not available) was used in the analysis. Phylloquinone intake (μg/d) was estimated using the National Cancer Institute Diet History Questionnaire administered at baseline and Diet∗Calc software. Other covariates missing or not measured in the 12-mo visit were imputed with values from the first clinic visit, as available.

### Statistical analyses

Plasma (dp)ucMGP concentration was categorized as <300 pmol/L (below the assay LLD), 300–449 pmol/L, and ≥450 pmol/L (the median concentration among those with detectable (dp)ucMGP) [24]. Plasma phylloquinone was categorized as <0.50 nmol/L, 0.50–0.99 nmol/L, or ≥1.00 nmol/L. These categories were based on the results of metabolic feeding studies that indicate plasma phylloquinone concentrations approximate 1.0 nmol/L when the recommended vitamin K adequate intakes are met [24, 28, 29]. Participant characteristics were compared across plasma (dp)ucMGP and phylloquinone categories using standard parametric and nonparametric approaches.

Poisson regression with robust variance estimation was used to determine the association between vitamin K status and CAC prevalence, incidence, and progression [30]. Robust Poisson regression models are alternative models for binary outcomes that have been shown to estimate risk ratios with less bias compared with logistic regression [31, 32]. Covariates included age, sex, education, race and ethnicity, BMI, eGFR, urine albumin, systolic and diastolic blood pressure (BP), triglycerides, LDL, HDL, diabetes, hypertension, statin use, CVD history, and smoking status and the first AS measure (in the progression models). Multiple linear regressions and linear mixed-effects models were used to evaluate differences in PWV across vitamin K status categories at baseline and over follow-up, with the follow-up visit as a fixed factor. Repeated measurements within subjects were modeled with compound symmetry covariance. Covariates included age, sex, education, race and ethnicity, BMI, estimated GFR, urine albumin, systolic and diastolic BP, heart rate, triglyceride, LDL, HDL, diabetes, hypertension, statin use, CVD history, and smoking status and first PWV measure (in linear-mixed models). Product terms were used to explore the effect modification by sex and race and ethnicity.

## Results

The baseline characteristics of the 2722 CRIC participants included in the analysis of CAC, PWV, or both are summarized in Table 1. Their mean (SD) age was 59 [11] y; 44% were women, 39% were non-Hispanic Black, 88% were hypertensive patients, and 48% reported type 2 diabetes. Plasma (dp)ucMGP was positively associated with age, hypertension, diabetes, CVD history, systolic BP, and triglycerides and inversely associated with estimated GFR and LDL and HDL cholesterols. Plasma phylloquinone was positively associated with triglycerides and inversely associated with HDL cholesterol and systolic BP (Table 1).

**Table 1:** Participant characteristics at baseline

Overall (*n* = 2722)		Plasma phylloquinone (nmol/L)^1^				Plasma (dp)ucMGP (pmol/L)^1^			
		<0.50 (*n* = 559)	0.50–1.00 (*n* = 909)	≥1.00 (*n* = 1251)	*P*	<300 (*n* = 1221)	300–450 (*n* = 906)	≥450 (*n* = 594)	*P*
Age (y)	59 ± 11	59 ± 11	58 ± 11	59 ± 11	0.66	58 ± 11	59 ± 11	60 ± 11	<0.001
Female sex, *n* (%)	1198 (44)	261 (47)	417 (46)	518 (41)	0.04	477 (39)	425 (47)	296 (50)	<0.001
Race and ethnicity, *n* (%)									
Non-Hispanic White	1216 (45)	201 (36)	413 (45)	602 (48)	<0.001	522 (43)	445 (49)	249 (42)	<0.001
Non-Hispanic Black	1062 (39)	252 (45)	356 (39)	451 (36)		556 (46)	312 (34)	193 (32)	
Hispanic	328 (12)	88 (16)	113 (12)	127 (10)		88 (7)	116 (13)	124 (21)	
Other	116 (4)	18 (3)	27 (3)	71 (6)		55 (5)	33 (4)	28 (5)	
Education,^2^*n* (%)									
Less than high school	489 (18)	137 (25)	155 (17)	195 (16)	<0.001	194 (16)	143 (16)	151 (25)	<0.001
High school graduate	481 (18)	98 (18)	166 (18)	216 (17)		188 (15)	175 (19)	118 (20)	
Some college	787 (29)	167 (30)	274 (30)	346 (28)		353 (29)	269 (30)	165 (28)	
College graduate or more	965 (35)	157 (28)	314 (35)	494 (39)		486 (40)	319 (35)	160 (27)	
BMI (kg/m^2^), *n* (%)									
<18.5	19 (1)	5 (1)	6 (1)	7 (1)	0.43	8 (1)	7 (1)	4 (1)	0.003
18.5–24.9	411 (15)	89 (16)	124 (14)	198 (16)		197 (16)	140 (15)	73 (12)	
25–29.9	847 (31)	174 (31)	302 (33)	370 (30)		394 (32)	260 (29)	193 (32)	
30–39.9	1116 (41)	216 (39)	365 (40)	534 (43)		504 (41)	385 (42)	227 (38)	
≥40	329 (12)	75 (13)	112 (12)	142 (11)		118 (10)	114 (13)	97 (16)	
Hypertension, *n* (%)	2394 (88)	504 (90)	806 (89)	1081 (86)	0.05	1048 (86)	797 (88)	548 (92)	<0.001
Diabetes, *n* (%)	1308 (48)	282 (50)	411 (45)	614 (49)	0.09	546 (45)	421 (46)	341 (57)	<0.001
Current smoker, *n* (%)	302 (11)	77 (14)	106 (12)	117 (9)	0.02	138 (11)	102 (11)	61 (10)	0.78
History of CVD, *n* (%)	852 (31)	173 (31)	274 (30)	403 (32)	0.58	353 (29)	277 (31)	221 (37)	0.001
Statin use, *n* (%)	1568 (58)	315 (56)	532 (59)	719 (57)	0.71	693 (57)	512 (57)	363 (61)	0.15
Systolic blood pressure (mm Hg)	126 ± 21	129 ± 23	127 ± 21	125 ± 20	<0.001	124 ± 20	126 ± 21	131 ± 23	<0.001
Diastolic blood pressure (mm Hg)	70 ± 13	70 ± 13	70 ± 13	70 ± 12	0.68	70 ± 12	70 ± 13	69 ± 13	0.26
Estimated GFR (mL/min/1.73 m^2^), *n* (%)									
<30	652 (24)	154 (28)	214 (24)	283 (23)	0.21	189 (15)	211 (23)	251 (42)	<0.001
30–44	881 (32)	185 (33)	295 (32)	400 (32)		370 (30)	298 (33)	213 (36)	
45–59	699 (26)	135 (24)	229 (25)	334 (27)		362 (30)	246 (27)	91 (15)	
≥60	490 (18)	85 (15)	171 (19)	234 (19)		300 (25)	151 (17)	39 (7)	
Urine albumin (mg/L), *n* (%)									
<30	1381 (51)	285 (51)	464 (51)	631 (50)	0.84	673 (55)	471 (52)	237 (40)	<0.001
30–299	742 (27)	143 (26)	250 (28)	347 (28)		317 (26)	250 (28)	174 (29)	
≥300	599 (22)	131 (23)	195 (21)	273 (22)		231 (19)	185 (20)	183 (31)	
Triglycerides (mg/dL),^2^*n* (%)									
<150	1698 (62)	444 (79)	616 (68)	636 (51)	<0.001	790 (65)	565 (62)	342 (58)	0.01
≥150	1024 (38)	115 (21)	293 (32)	615 (49)		431 (35)	341 (38)	252 (42)	
HDL (mg/dL)^2^	49 ± 16	50 ± 16	49 ± 15	48 ± 16	0.004	49 ± 15	49 ± 16	46 ± 15	<0.001
LDL (mg/dL)^2^	101 ± 35	98 ± 33	102 ± 34	102 ± 36	0.07	102 ± 34	103 ± 35	97 ± 35	0.003
Heart rate (bpm)^2^	65 ± 11	66 ± 12	65 ± 11	64 ± 11	0.10	64 ± 11	65 ± 11	66 ± 12	0.02
Agatston score^2^^,^^3^	28 (281)	27 (243)	19 (263)	41 (326)	0.19	13 (213)	45 (413)	59 (354)	<0.001
Phylloquinone intake (μg/d)^2^^,^^3^	116 (139)	106 (105)	116 (135)	122 (164)	<0.001	123 (172)	110 (118)	112 (122)	0.009

1 Data are presented as mean ± SD with the ANOVA test for numeric data and count (%) with the χ^2^ test for categorical data, unless indicated otherwise. There are 3 values missing in plasma phylloquinone and 1 missing in (dp)ucMGP.

2 There is 1 missing value each in education, HDL, LDL, and triglycerides. Among 2534 participants with PWV measures, heart rates were missing in 7. Phylloquinone intake was measured in 2286 participants. Agatston score was measured in 1774 participants.

3 The baseline Agatston score and phylloquinone intake are presented as median (IQR) and tested by the Kruskal–Wallis test. (dp)ucMGP, dephospho-uncarboxylated matrix gla protein; PWV, pulse wave velocity.

**Figure 1:**
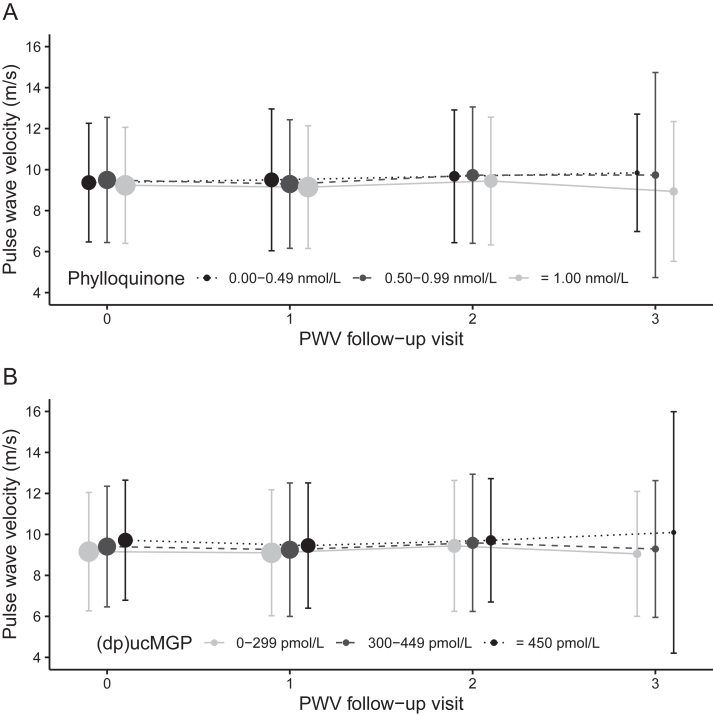
Longitudinal association between vitamin K status and arterial stiffness in the Chronic Renal Insufficiency Cohort study. Pulse wave velocity (PWV) according to (A) plasma phylloquinone and (B) plasma dephospho-uncarboxylated matrix gla protein [(dp)ucMGP] by visit. No significant time interaction was observed [*P* = 0.40 for phylloquinone × visit interaction and *P* = 0.63 for (dp)ucMGP × visit interaction]. Data are cell means at visit 0 and LS means at visits 1–3. Error bars are the SDs. Models to generate LS means are adjusted for age, sex, education, race and ethnicity, BMI, estimated GFR, systolic and diastolic blood pressure (BP), triglyceride, HDL, LDL, diabetes, hypertension, statin use, CVD history, smoking status, heart rate, first PWV measure, and urine albumin. Sample size in each group is reflected by the size of the symbol. For phylloquinone, *n* = 1834 at visit 0, *n* = 1834 at visit 1, *n* = 628 at visit 2, and *n* = 136 at visit 3. For (dp)ucMGP, *n* = 1835 at visit 0, *n* = 1835 at visit 1, *n* = 629 at visit 2, and *n* = 136 at visit 3.

Overall, 88% of the participants had detectable CAC at baseline. However, the prevalence of CAC did not differ significantly according to plasma phylloquinone or plasma (dp)ucMGP (Table 2). Among the participants without detectable CAC at baseline, the incidence of CAC (defined as AS > 0 at follow-up) did not significantly differ according to plasma phylloquinone or plasma (dp)ucMGP concentrations (Table 2). Moreover, the incidence rate for CAC progression did not significantly differ according to plasma phylloquinone concentrations (Table 2). Among the participants with detectable CAC at baseline, those with 300–449 pmol/L plasma (dp)ucMGP concentration had a 49% lower rate of CAC progression than participants with ≥450 pmol/L. However, the incidence rate ratio for CAC progression did not differ significantly between those with <300 pmol/L and those with ≥450 pmol/L plasma (dp)ucMGP concentrations. The association of vitamin K status biomarkers with CAC prevalence did not differ by sex or race and ethnicity (all *P*-interaction > 0.38). After adjustment for pertinent confounders, neither biomarker of vitamin K status was associated with PWV, either cross-sectionally or longitudinally (Figure 1 and Table 3). The associations of plasma phylloquinone and (dp)ucMGP with PWV were similar in men and women cross-sectionally and longitudinally (all *P*-interaction > 0.12). We detected a significant interaction between plasma (dp)ucMGP and race and ethnicity regarding the cross-sectional with PWV (*P*-interaction = 0.01) (Supplemental Table 1), but the longitudinal association did not differ by race and ethnicity (*P*-interaction = 0.84), nor did the association of plasma phylloquinone with PWV differ by race and ethnicity (*P*-interaction > 0.14).

**Table 2:** Association of vitamin K status with CAC prevalence, incidence, and progression

CAC prevalence – defined as AS > 0 at baseline. Data are PR (95% CI) based on Poisson regression				
Plasma (dp)ucMGP	*n*	<300 pmol/L	300–449 pmol/L	≥450 pmol/L
Unadjusted	1774	0.87 (0.79, 0.94)	0.98 (0.90, 1.06)	Reference
Fully adjusted^1^	1774	0.96 (0.88, 1.04)	1.02 (0.95, 1.11)	Reference
Plasma phylloquinone		<0.50 nmol/L	0.50–0.99 nmol/L	≥1.00 nmol/L
Unadjusted	1771	Reference	0.96 (0.87, 1.06)	1.03 (0.94, 1.12)
Fully adjusted^1^	1771	Reference	0.99 (0.90, 1.08)	1.01 (0.93, 1.10)
CAC incidence – defined as AS > 0 at follow-up among people without CAC at baseline. Data are IRR (95% CI).				
Plasma (dp)ucMGP	*n*	<300 pmol/L	300-449 pmol/L	≥450 pmol/L
Unadjusted	394	0.76 (0.48, 1.18)	0.66 (0.39, 1.10)	Reference
Fully adjusted^1^	394	1.30 (0.77, 2.19)	1.01 (0.61, 1.66)	Reference
Plasma phylloquinone		<0.50 nmol/L	0.50–0.99 nmol/L	≥ 1.00 nmol/L
Unadjusted	394	Reference	0.78 (0.47, 1.29)	0.96 (0.61, 1.54)
Fully adjusted^1^	394	Reference	0.90 (0.54, 1.50)	1.21 (0.76, 1.91)
CAC progression – defined as annual increase ≥100 AU among people with CAC at baseline. Data are IRR (95% CI).				
Plasma (dp)ucMGP	*n*	<300 pmol/L	300–449 pmol/L	≥450 pmol/L
Unadjusted	622	0.60 (0.41, 0.89)	0.52 (0.34, 0.79)	Reference
Fully adjusted^1^	622	0.82 (0.56, 1.19)	0.51 (0.33, 0.78)	Reference
Plasma phylloquinone		<0.50 nmol/L	0.50–0.99 nmol/L	≥1.00 nmol/L
Unadjusted	621	Reference	1.43 (0.83, 2.47)	1.50 (0.91, 2.50)
Fully adjusted^1^	621	Reference	1.71 (0.98, 3.00)	1.50 (0.89, 2.51)

1 Fully adjusted model: adjusted for age, sex, education, race and ethnicity, BMI, estimated GFR, systolic and diastolic blood pressure, triglyceride, HDL, LDL, diabetes, hypertension, statin use, CVD history, smoking status, urine albumin, and baseline AS (in progression model). AS, Agatston score; AU, Agatston unit; CAC, coronary artery calcium; (dp)ucMGP, dephospho-uncarboxylated matrix gla protein; IRR, incidence rate ratio; PR, prevalence ratio.

**Table 3:** Cross-sectional association between vitamin K status and PWV

	Unadjusted		Fully adjusted^1^	
	LS mean (95% CI) PWV	*P*	LS mean (95% CI) PWV	*P*
Plasma phylloquinone (nmol/L), *n* = 2525				
<0.50	9.11 (8.87, 9.35)	Reference	8.98 (8.70, 9.27)	Reference
0.50–1.00	9.15 (8.97, 9.34)	0.77	9.21 (8.95, 9.48)	0.07
≥1.00	9.04 (8.89, 9.20)	0.66	9.00 (8.76, 9.24)	0.91
Plasma (dp)ucMGP (pmol/L), *n* = 2526				
<300	8.88 (8.73, 9.04)	<0.001	9.02 (8.77, 9.27)	0.71
300–450	9.12 (8.94, 9.31)	0.010	9.11 (8.86, 9.37)	0.70
≥450	9.52 (9.28, 9.76)	Reference	9.06 (8.79, 9.35)	Reference

1 Models were adjusted for age, sex, education, race and ethnicity, BMI, estimated GFR, systolic and diastolic blood pressure, triglyceride, HDL, LDL, diabetes, hypertension, statin use, CVD history, smoking status, heart rate, and urine albumin. (dp)ucMGP, dephospho-uncarboxylated matrix gla protein; LS, least square; PWV, pulse wave velocity.

## Discussion

In a large and diverse cohort of people with CKD, we found no consistent differences in CAC prevalence, incidence, or progression across categories of vitamin K status. CAC reflects subclinical atherosclerotic CVD. Given our recent findings that atherosclerotic CVD risk did not differ across plasma phylloquinone categories in CRIC [24], it was not surprising that plasma phylloquinone too was not associated with CAC. We also found no difference in CAC prevalence or incidence across plasma (dp)ucMGP categories. Our results are generally consistent with the available recent evidence from vitamin K supplementation randomized controlled trials (RCTs), which collectively do not support a protective role for vitamin K supplementation in improving the vascular health of individuals with CKD [[33], [34], [35]].

It should be noted that although there were no differences in CAC prevalence, incidence, or progression across categories of plasma phylloquinone, among participants with pre-existing CAC, those with 300–450 pmol/L plasma (dp)ucMGP concentration had a 49% lower rate of CAC progression than in those with plasma (dp)ucMGP concentration of ≥450 pmol/L. However, the rate of CAC progression did not differ between those with <300 pmol/L and ≥450 pmol/L plasma (dp)ucMGP concentrations. Plasma (dp)ucMGP concentration decreases as vitamin K status increases, so the lowest progression rate would be expected in those with the lowest (dp)ucMGP. We do not have a ready explanation for the observed U-shaped association between plasma (dp)ucMGP concentration and CAC progression. Because the risk for atherosclerotic CVD events did not differ according to plasma (dp)ucMGP concentration in CRIC [24], nor did the prevalence or incidence of CAC, we cannot discount the possibility this observation was due to chance.

Similarly, neither plasma phylloquinone nor (dp)ucMGP concentrations were associated with PWV cross-sectionally or over the follow-up. The previous studies of vitamin K status and arterial stiffness in CKD used (dp)ucMGP as the only biomarker of vitamin K status and were cross-scetional. Plasma (dp)ucMGP was positively correlated with PWV in a small study of African Americans on hemodialysis (*n* = 37) [13] and positively predicted PWV in 137 adults (55% White), more than half of whom had mildly or moderately reduced kidney function [14]. Conversely, plasma (dp)ucMGP was not associated with PWV or CAC in 391 primarily African-American individuals on hemodialysis [10]. Arterial stiffness is associated with heart failure and atrial fibrillation [36, 37], and we have reported that high plasma (dp)ucMGP was associated with a higher risk of heart failure and atrial fibrillation in CRIC [24]. However, the findings of this study suggest this association is unrelated to arterial stiffness.

In a systematic review that included 6 RCTs that evaluated the effect of vitamin K on arterial calcification, the collective evidence did not support vitamin K supplementation in reducing arterial calcification in community- and clinic-based populations [33]. In a subsequent RCT of diabetics with a baseline AS ≥ 10 AUs [median (IQR): 180 (62–502) AU], phylloquinone supplementation (10 mg/d) for 3 mo showed no effect on coronary calcification activity measured using 18F-NaF positron emission tomography [38]. However, in a post hoc analysis of the same trial, the investigators reported phylloquinone-supplemented participants had significantly lower odds of developing new calcifying lesions [39]. A trial conducted in adults with stage 3b-4 CKD found no effect of 400 μg/d menaquinone-7 (a form of vitamin K2) on arterial stiffness or other measures of vascular health over 1 y [34]. In a recently completed pilot RCT comprising individuals on hemodialysis with a baseline AS of ≥30 AU, phylloquinone supplementation (10 mg thrice weekly) showed no effect on CAC progression over 1 y [35]. Results of RCTs focused on arterial stiffness were similarly unconvincing [33]. However, in many of these trials, vitamin K supplementation significantly lowered plasma (dp)ucMGP concentration [33, 34, 40]. In the absence of any physiological corollary to change in dp(uc)MGP in this patient population [33, 34], the merits of this biomarker as a proxy for vascular health is elusive.

This study is strengthened by the large and diverse cohort of individuals with CKD, use of gold-standard techniques to measure CAC and PWV, and use of 2 biomarkers of vitamin K status. Moreover, the longitudinal component was a notable strength. Cross-sectional studies cannot account for possible reverse-causation effects, which may be involved in the reported positive associations between plasma (dp)ucMGP concentration and arterial stiffness [13, 14, 41, 42]. Increased BP, cardiac remodeling, and heart failure, which are associated with arterial stiffening, can upregulate MGP expression [[43], [44], [45]]. MGP expression can influence the amount of (dp)ucMGP in circulation, independent of vitamin K status. Hence, positive cross-sectional associations between (dp)ucMGP concentration and arterial stiffness may reflect associations between cardiac changes that influence MGP expression, regardless of vitamin K status, and that are associated with arterial stiffening.

The study’s limitations merit consideration. Because both vitamin K status biomarkers were measured at 1 time point, we were unable to account for possible status changes during the follow-up period. Plasma (dp)ucMGP concentration should be corrected for total MGP to more accurately reflect vitamin K status [46] [as is typically done with other vitamin K–dependent proteins [47]]. However, we were unable to adjust our models or express (dp)ucMGP as a ratio of total MGP because total MGP was not measured in CRIC. Because a threshold defining high plasma (dp)ucMGP concentration has not been established, data-driven categories were used, which are only directly relevant to the sample from which they were derived [48]. Plasma (dp)ucMGP concentration was lower in CRIC, suggestive of a better vitamin K status, than in other studies of individuals with CKD that measured plasma (dp)ucMGP concentration using the same assay we used [10, 13, 49], which may have influenced our findings. However, given the size and diversity of the CRIC cohort, it is more broadly representative of individuals with CKD in the United States. That being said, our findings are not generalizable to warfarin users because they were excluded because warfarin is a vitamin K antagonist. However, because high vitamin K intake can interfere with the effectiveness of warfarin, it is unlikely that warfarin users would be included in vitamin K supplementation trials.

In summary, vitamin K status was generally not associated with coronary calcification or arterial stiffness in adults with mild-to-moderate CKD. Moreover, the collective results of the available RCTs do not support a protective role for vitamin K supplementation in improving the vascular health of individuals with CKD [[33], [34], [35]]. Given the burden of poor vascular health in CKD, alternate therapeutic strategies merit investigation.

## Funding

This project was supported by the National Institute of Diabetes and Digestive and Kidney Diseases (R01DK111392 to MKS) and the USDA Agricultural Research Service Cooperative Agreement 58-8050-9-004. Funding for the Chronic Renal Insufficiency Cohort Study was provided by co-operative agreements from the National Institute of Diabetes and Digestive and Kidney Diseases (U01DK060990, U01DK060984, U01DK061022, U01DK061021, U01DK061028, U01DK060980, U01DK060963, and U01DK060902). In addition, this work was supported in part by the University of Pennsylvania Clinical and Translational Research Center (UL1 RR-024134) and NIH/National Center for Advancing Translational Science (NCATS) (UL1TR000003), Johns Hopkins University (UL1 RR-025005 and UL1 TR-000424), University of Maryland General Clinical Research Center (M01 RR-16500), Clinical and Translational Science Collaborative of Cleveland (UL1TR000439), the NIH/NCATS and NIH roadmap for Medical Research, Michigan Institute for Clinical and Health Research (UL1RR024986), University of Illinois at Chicago Center for Clinical and Translational Sciences (UL1RR029879), Tulane University Translational Research in Hypertension and Renal Biology (P30GM103337), and Kaiser NIH/National Center for Research Resources, University of California, San Fransisco, Clinical and Translational Science Institute (UL1 RR-024131). The sponsors had no role in the design and conduct of the study, collection, management, analysis, and interpretation of the data; preparation, review, or approval of the manuscript; and decision to submit the manuscript for publication. The content is the sole responsibility of the authors and does not necessarily represent the official views of the NIH or the USDA.

## Author disclosures

KB reports additional grant support from NIH and honoraria for NIH and Department of Health and Human Services grant reviews and for serving as a member of the American Society of Nutrition Statistical Review Board. SLB serves as Vice President elect and Board member of the American Society of Nutrition. HIF serves on Steering Committees for the Chronic Renal Insufficiency Cohort Study and the NIH-NIDDK and on the National Kidney Foundation (NKF) Advisory Board; reports consultancy agreements with Essure Litigation (DLA Piper LLP), InMed, Inc., Kyowa Hakko Kirin Co, Ltd., and NKF; and receives honoraria from the University of California Los Angeles (invited speaker) and Rogosin Institute (invited speaker); and serves as the Editor-in-Chief of the American Journal of Kidney Disease. RT reports consultancy agreements with Medtronic, serving on the Data Safety Monitoring Board for AXIO and receiving royalties as an UpToDate contributor. SR reports additional grant support from NIH, Bayer, and AstraZeneca; honoraria from the American Diabetes Association and NKF; and advisory/consultancy roles for Bayer, Relypsa, Reata, and Teladoc. DEW reports receiving support to his institution from Dialysis Clinic, Inc. All other authors report no conflicts of interest. At the time of submission, SB was a Deputy Editor for Current Developments in Nutrition and KB was a Statistical Editor for the American Society of Nutrition. Neither of them were involved in the journal’s evaluation of this manuscript.

## Data Availability

The data described in the manuscript, code book, and analytic code will be made available on request pending approval of the CRIC Study Steering Committee.
