# Supplementary material for: Association of Vitamin K Status with Arterial Calcification and Stiffness in Chronic Kidney Disease: The Chronic Renal Insufficiency Cohort
Source: Curr Dev Nutr. 2022 Dec 23;7(1):100008. doi: 10.1016/j.cdnut.2022.100008 (PMC10100935; doi:10.1016/j.cdnut.2022.100008)
Supplement: Multimedia component 1 [file mmc1.docx]

# **
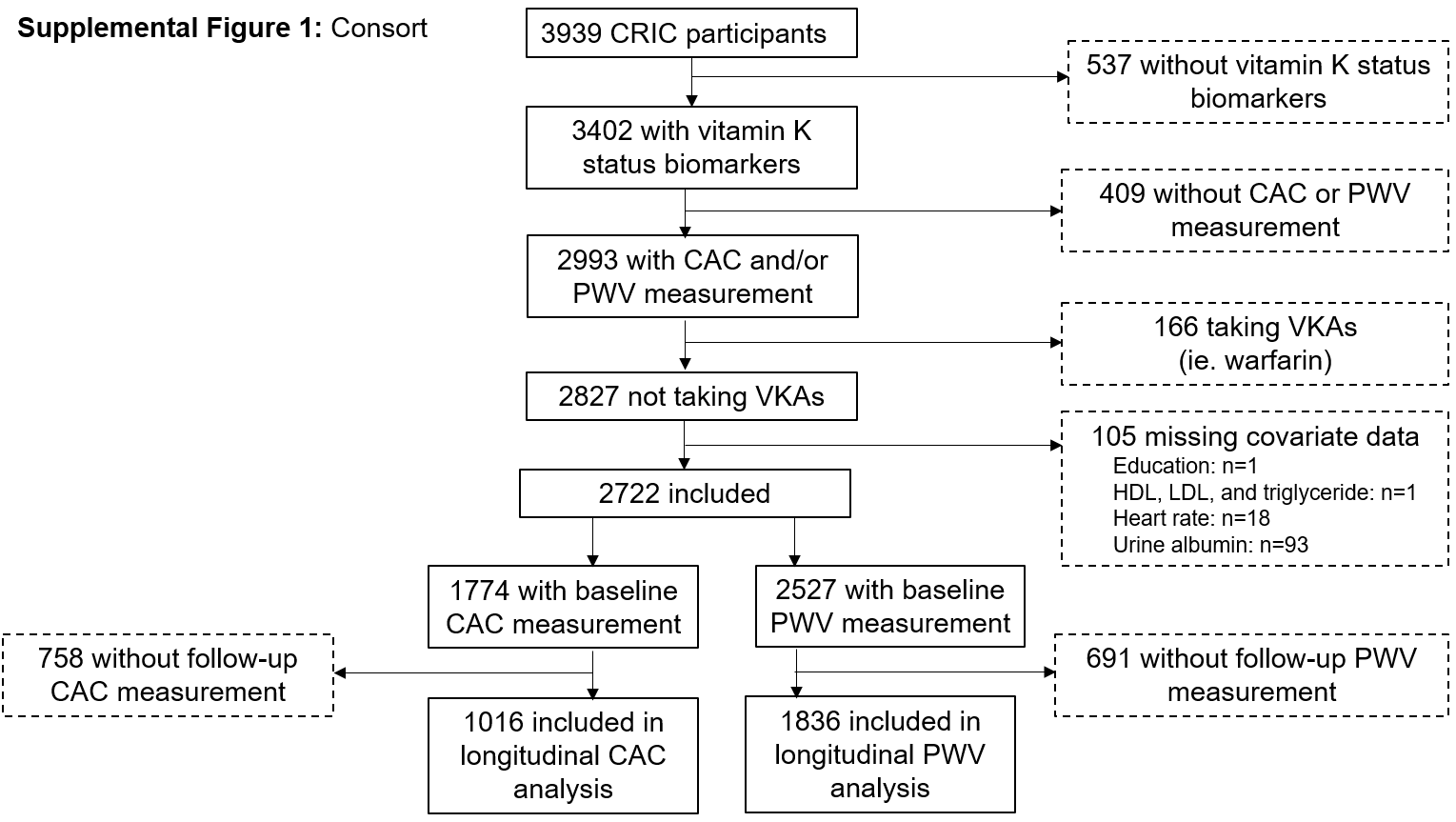
**

# **Supplemental Table 1:** Cross-sectional differences in PWV across plasma (dp)ucMGP categories overall and in race-ethnic subgroups

|  |  | plasma (dp)ucMGP | | |
| --- | --- | --- | --- | --- |
|  | n | <300 pmol/L | 300-450 mol/L | ≥ 450 pmol/L |
| Non-Hispanic white | 1141 | 8.95 (8.60, 9.31) | 9.17 (8.82, 9.54) | 9.45 (9.05, 9.87) |
| Non-Hispanic black | 979 | 9.84 (9.38, 10.32) | 9.77 (9.28, 10.29) | 9.38 (8.84, 9.95) |
| Hispanic | 300 | 8.67 (7.84, 9.59) | 8.54 (7.72, 9.44) | 8.58 (7.79, 9.45) |
| Other | 106 | 9.11 (7.70, 10.79) | 9.33 (8.06, 10.79) | 8.79 (7.50, 10.30) |
| Data are least squares means (95%CI).  Models were adjusted for age, sex, education, race & ethnicity, BMI, eGFR, systolic and diastolic BP, triglyceride, HDL, LDL, diabetes, hypertension, statin use, CVD history, smoking status, heart rate, and urine albumin. | | | | |
